# Hospital Administration

**Published:** 1892-07-23

**Authors:** 


### HOSPITAL ADMINISTRATION.

### III.—FINANCE.

The primary object of all hospital administration is to further the measures by which the institution may be maintained free from pecuniary obligations. From the voluntary point of view, judging from the advertisement columns of the newspapers, this condition is far from satisfactory, involving incessant appeals to propitiate public charity, which are not always responded to in the way they deserve to be. To forward the demand for assistance the hospital has to appear as if in a state of chronic impecuniosity, and its administrative authorities are driven to adopt numerous expedients of raising money which, under other circumstances, might be considered questionable. One of the necessary effects of the voluntary system is to engender a species of rivalry among the charities as to which can prove itself most worthy of support, and the prize naturally falls to the lot of those most successful in enlisting public sympathy by their works, and by making them universally known. On the other hand, there is a singular aversion on the part of most of the authorities of voluntary hospitals to receiving money from the State, lest they should come under, in a manner however partial, its jurisdiction and control. Judging from these facts, there is every probability that in future the Local Government, which has already outstripped the voluntary hospitals and infirmaries in the accommodation which it has provided for the sick poor will continue to do so, especially in the departments of chronic, incurable and infectious diseases, from which it has already gratefully relieved the hospitals.

To throw light on the economic administration and further the unity among hospitals, few things would prove so advantageous as the general adoption of a uniform system of accounts. The want of this has long been felt in the hospital world, and for some time past some of the most able secretaries have been engaged in concocting model plans by which the desideratum may be obtained, and it is to be hoped that their exertions will not go unrewarded. At the same time, the force of the voluntary system consists in its freedom from restraint and interference, and if hospital committees are satisfied with their systems of keeping accounts it is highly improbable that they would be disposed to alter them. One obvious result of uniformity of this nature would be an easy means of comparing the expenditure in different departments of one hospital with another, and the proportion which this bears to the amount of relief afforded. Various attempts are made in this way to determine the cost of beds continuously occupied in various hospitals, and although all are more or less subject to some objection, particularly with regard to what may be considered ordinary as opposed to extraordinary expenditure, they nevertheless afford, when pursued for several successive years, a very fair standard of comparison to judge economic management or the reverse. The process giving the most trustworthy results is arrived at by simply dividing the total annual expenditure, exclusive o new buildings, and interest on debt, if any, by the average number of patients in daily residence during the same year, disregarding altogether the number and description of out-patients. The omission of the latter is necessary to remove from the calculation a disturbing element which interferes materially with attempts to arrive even at an approximate estimate, as is shown by the discrepancies in the alleged cost of out-patients in the metropolitan hospitals, where they are seen to range from a minimum of 6d. to a maximum of 25s. each.* Estimated by the above process the annual cost of a bed in most of the leading hospitals in Great Britain and Ireland may be judged of by the following figures, taken from "Burdett's Hospital Annual," 1891-92.

| Name of Hospital. | Beds in occupation. | Average number occupied. | Total admitted. | Average cost per occupied bed. | Cost of each patient. |
|---|---|---|---|---|---|
| The London | 790 | 622 | 8,503 | 95 10 8 | 5 12 0 |
| St. Bartholomew's | 667 | 534 | 6,765 | 121 13 9 | 8 16 6 |
| Guy's | 508 | 432·7 | 5,710 | 88 9 9 | 6 14 1 |
| St. Thomas's | 436 | 365 | 4,699 | 103 9 6 | 7 15 3 |
| St. George's | 356 | 316 | 4,265 | 86 11 6 | 6 2 1 |
| Middlesex | 310 | 254 | 2,914 | 112 4 0 | 7 16 6 |
| St. Mary's | 281 | 251 | 3,451 | 92 15 5 | 5 19 11 |
| University | 207 | 179 | 3,007 | 105 19 7 | 5 13 3 |
| King's | 206 | 163 | 2,033 | 106 3 4 | 8 0 7 |
| Westminster | 205 | 172 | 2,572 | 76 0 2 | 4 11 1 |
| Charing Cross | 175 | 141 | 1,948 | 106 4 4 | 7 2 9 |
| Royal Free | 150 | 133 | 2,019 | 76 0 8 | 4 8 1 |
| Lon. Homœopathic | 94 | 55 | 830 | 84 2 10 | 4 19 0 |
| **Some Provincial General Hospitals** | | | | | |
| Leeds Infirmary | 320 | 290 | 4,814 | 57 7 4 | 3 2 9 |
| Manchester Royal | 298 | 263 | 4,239 | 61 15 2 | 3 8 10 |
| Birmingham | 280 | 213 | 3,607 | 71 4 0 | 3 8 2 |
| Newcastle-on-Tyne | 270 | 242 | 3,120 | 50 5 11 | 3 14 10 |
| Bristol Royal | 264 | 209 | 3,139 | 66 12 1 | 3 6 4 |
| Sheffield | 200 | 160 | 1,713 | 61 5 5 | 5 4 0 |
| Liverpool Royal | 200 | 170 | 1,975 | 44 12 7 | 3 12 9 |
| Cambridge | 152 | 119 | 1,228 | 50 15 9 | 4 13 9 |
| Oxford | 138 | 99 | 1,310 | 71 17 5 | 5 4 1 |
| Edinburgh Royal | 700 | 634 | 8,068 | 56 13 0 | 4 5 3 |
| Aberdeen Royal | 213 | 160 | 2,131 | 37 3 7 | 2 15 5 |
| Glasgow Royal | 582 | 538 | 5,408 | 50 5 7 | 4 14 8 |
| Glasgow Western | 400 | 371 | 3,716 | 51 1 7 | 4 18 8 |
| Dublin (Steeven's) | 230 | 82 | 1,164 | 70 12 2 | 4 6 0 |
| Adelaide Hospital | 125 | 82 | 861 | 79 16 10 | 6 8 10 |
| Belfast Hospital | 189 | 135 | 2,223 | 50 18 1 | 2 9 10 |
| **Statistics of Some Special London Hospitals.** | | | | | |
| Brompton Cnsmpt. | 337 | 275 | 1,667 | 87 14 6 | 14 0 9 |
| Royal, Dis. of Chst. | 80 | 37 | 358 | 140 9 10 | 13 9 1 |
| Centrl. Thrt. & Ear | 16 | 12 | 218 | 164 9 10 | 7 12 10 |
| Children's, Great Ormond Street | 127 | 92 | 733 | 109 15 10 | 12 9 9 |
| Childrn's, N.-Estn. | 55 | 37 | 531 | 136 13 7 | 8 3 0 |
| Samaritan Free | 54 | 51 | 520 | 109 19 1 | 10 6 5 |
| Grosvenor, Women and Children | 14 | 11 | 103 | 101 8 0 | 9 14 11 |
| Royl. Lon. Opthmic. | 100 | 96 | 2,337 | 67 13 2 | 2 4 1 |
| London Fever | 220 | 54 | 471 | 137 19 11 | 15 16 4 |
| National, Paralysed | 175 | 148 | 747 | 73 3 6 | 13 18 9 |
| Cancer Hospital | 100 | 80 | 679 | 104 14 0 | 12 5 1 |
| **Some Special Provincial Hospitals.** | | | | | |
| Manchester Sick Children | 140 | 117 | 1,180 | 62 0 7 | 5 14 7 |
| Birmingham ditto | 74 | 52 | 654 | 83 6 8 | 5 11 6 |
| Derby ditto | 41 | 30 | 316 | 22 6 8 | 1 16 0 |
| Aberdeen ditto | 80 | 56 | 428 | 28 8 5 | 3 12 4 |
| Edinburgh ditto | 76 | — | 623 | — | 5 0 1 |
| Glasgow ditto | 70 | 61 | 566 | 55 18 5 | 5 13 2 |
| Bristol ditto | 102 | 88 | 807 | 39 3 6 | 4 0 8 |
| Exeter Eye Infirmary | 50 | 33 | 257 | 27 15 1 | 3 5 2 |
| Glasgow ditto | 112 | 62 | 1,165 | 49 6 6 | 2 2 9 |
| Dublin, St. Mark's Eye Infirmary | 50 | 43 | 717 | 35 1 0 | 1 16 3 |
| Dublin Eye or Ear Infirmary | 30 | 23 | 362 | 38 15 6 | 2 2 5 |

It may be quite possible for those best acquainted with the internal arrangements of the various hospitals referred to in the tables to account for the remarkable differences which exist in their relative cost in affording medical relief, by pointing out peculiarities associated with the administration

* Statistics for the use of the Council, Metropolitan Hospitals' Sunday Fund.

of each institution. Hospitals which are not only medical schools but also nurse-training establishments are usually, though not always, conducted at greater expense than others not so incorporated. Others again, more liberal than the majority, pay their medical staff certain gratuities for their services, and find it necessary to keep abreast of the time in all sanitary improvements, including continuous repairs to the fabric, the increase and development of the nursing staff, and by contributing to the comforts of the sick in a variety of ways which a past generation hardly dreamt of. The discrepencies observable in the comparative cost of the special hospitals are still more apparent, and here it may be fairly attributed to the paucity of beds in proportion to a large and expensive out-patient service. One striking feature in these returns is the reduced cost at which patients may be maintained in provincial hospitals when compared with London, a fact which can only be explained by their being fed, nursed, and doctored in a more sumptuous manner in the metropolis than anywhere else. Something, however, must be allowed for the greater expense of the out-patients service of the metropolitan hospitals, where it has undergone a greater development and formed such a useful adjunct to the general hospital, as a school of instruction as well as an important curative agency.

